# Seroprevalence and risk factors for SARS-CoV-2 among incarcerated adult men in Quebec, Canada (2021)

**DOI:** 10.1093/cid/ciac031

**Published:** 2022-01-17

**Authors:** Nadine Kronfli, Camille Dussault, Mathieu Maheu-Giroux, Alexandros Halavrezos, Sylvie Chalifoux, Jessica Sherman, Hyejin Park, Lina Del Balso, Matthew P Cheng, Sébastien Poulin, Joseph Cox

**Affiliations:** Centre for Outcomes Research and Evaluation, Research Institute of the McGill University Health Centre, Montréal, Québec, Canada; Department of Medicine, Division of Infectious Diseases and Chronic Viral Illness Service, McGill University Health Centre, Montréal, Québec, Canada; Centre for Outcomes Research and Evaluation, Research Institute of the McGill University Health Centre, Montréal, Québec, Canada; Department of Epidemiology and Biostatistics, School of Population and Global Health, Faculty of Medicine and Health Sciences, McGill University, Montréal, Québec, Canada; Centre for Outcomes Research and Evaluation, Research Institute of the McGill University Health Centre, Montréal, Québec, Canada; Centre for Outcomes Research and Evaluation, Research Institute of the McGill University Health Centre, Montréal, Québec, Canada; Centre for Outcomes Research and Evaluation, Research Institute of the McGill University Health Centre, Montréal, Québec, Canada; Centre for Outcomes Research and Evaluation, Research Institute of the McGill University Health Centre, Montréal, Québec, Canada; Centre for Outcomes Research and Evaluation, Research Institute of the McGill University Health Centre, Montréal, Québec, Canada; Department of Medicine, Divisions of Infectious Diseases and Medical Microbiology, McGill University Health Centre, Montréal, Québec, Canada; Centre intégré de santé et de services sociaux des Laurentides, Saint-Jérôme, Québec, Canada; Centre for Outcomes Research and Evaluation, Research Institute of the McGill University Health Centre, Montréal, Québec, Canada; Department of Medicine, Division of Infectious Diseases and Chronic Viral Illness Service, McGill University Health Centre, Montréal, Québec, Canada; Department of Epidemiology and Biostatistics, School of Population and Global Health, Faculty of Medicine and Health Sciences, McGill University, Montréal, Québec, Canada

**Keywords:** SARS-CoV-2, seroprevalence, antibody, incarceration, prison

## Abstract

**Background:**

People in prison are at increased risk of SARS-CoV-2 infection due to overcrowding and challenges in implementing infection prevention and control measures. We examined the seroprevalence of SARS-CoV-2 and associated carceral risk factors among incarcerated adult men in Quebec, Canada.

**Methods:**

We conducted a cross-sectional seroprevalence study in 2021 in three provincial prisons, representing 45% of Quebec’s incarcerated male provincial population. The primary outcome was SARS-CoV-2 antibody seropositivity (Roche Elecsys® serology test). Participants completed self-administered questionnaires on sociodemographic, clinical, and carceral characteristics. The association of carceral variables with SARS-CoV-2 seropositivity was examined using Poisson regression models with robust standard errors. Crude and adjusted prevalence ratios (aPR) with 95% confidence intervals (95%CI) were calculated.

**Results:**

Between January 19 and September 15, 2021, 246 of 1,100 (22%) recruited individuals tested positive across three prisons (range 15–27%). Seropositivity increased with time spent in prison since March 2020 (aPR 2.17, 95%CI 1.53–3.07 for “*all*” vs. “*little time*”), employment during incarceration (aPR 1.64, 95%CI 1.28–2.11 vs. not), shared meal consumption during incarceration (“*with cellmates*”: aPR 1.46, 95%CI 1.08–1.97 vs. “*alone*”; “*with sector*”: aPR 1.34, 95%CI 1.03–1.74 vs. “*alone*”), and incarceration post-prison outbreak (aPR 2.32, 95% CI 1.69–3.18 vs. “*pre-outbreak”*).

**Conclusions:**

The seroprevalence of SARS-CoV-2 among incarcerated individuals was high and varied between prisons. Several carceral factors were associated with seropositivity, underscoring the importance of decarceration and occupational safety measures, individual meal consumption, and enhanced infection prevention and control measures including vaccination during incarceration.

**Introduction**

Canadian correctional settings have witnessed several large outbreaks of novel coronavirus (SARS-CoV-2) since the start of the pandemic [1,2]. Overcrowding, poor ventilation and unsanitary conditions, limited testing, and challenges in accessing and implementing effective infection prevention and control measures [3-7] have accelerated transmission of SARS-CoV-2 among those living in correctional facilities, resulting in levels of transmission that are several-fold higher than most surrounding communities [3,6,8]. An aging and comorbid Canadian carceral population [9] and the disproportionate incarceration of people experiencing social and health inequities [10,11] underscore the importance of implementing prison-based preventative measures to mitigate future outbreaks in correctional settings.

In Canada, an estimated 38,000 people are incarcerated each day – 14,000 in federal custody and 24,000 in provincial/territorial custody [9]. Since the start of the COVID-19 pandemic, the average daily incarcerated provincial population in Quebec (approximately 4,500 individuals) was reduced by 20% – a deliberate preventative measure to reduce prison crowding through decreased justice system activities (e.g., postponed trials, fewer arrests, etc.), lower incarceration rates, and the early release of low-risk individuals [2,12,13]. Furthermore, the *Canadian National Advisory Committee on Immunization* (NACI) prioritized "resident and staff of congregate settings" for early COVID-19 vaccination in December 2020 [14]. However, COVID-19 vaccine roll-out for people incarcerated in provincial prisons, including Quebec, has trailed the federal response by *Correctional Service Canada* [2,15,16]. With the delta variant, *maintaining* prison-based preventative measures until high vaccine coverage is achieved remains crucial, particularly with high prison-based transmission [7].

While over 750 people incarcerated in Quebec's provincial prisons have tested positive for SARS-CoV-2 since March 2020 [2] – the start of the COVID-19 pandemic in Quebec – this likely underestimates the true extent of SARS-CoV-2 exposure in provincial prisons. Several reasons likely contribute including decreased disclosure of symptoms due to the mandatory quarantine or isolation of those who test SARS-CoV-2 PCR positive, a focus on symptom-based testing [6], logistical difficulties of SARS-CoV-2 testing in prison settings, and the high turnover of those incarcerated in provincial prisons [9]. To reduce SARS-CoV-2 transmission, a mandatory 14-day isolation on admission, the provision of masks, and the cessation of inter-facility transfers were introduced. Seroprevalence studies have been conducted in correctional settings in low- and middle-income countries [17,18], where limited mitigation strategies likely contribute to higher SARS-CoV-2 seroprevalence. Therefore, we examined the seroprevalence of SARS-CoV-2 antibodies among people incarcerated in Quebec provincial prisons, and determined the effects of carceral exposures on SARS-CoV-2 seropositivity.

## Methods

### Study design and setting

We conducted an observational cross-sectional study in three correctional facilities under the responsibility of the *Ministère de la sécurité publique du Québec* (MSP). The MSP oversees provincial corrections, where adult individuals serve sentences of less than two years [9]. Three large provincial correctional facilities, representing 45% of the incarcerated male provincial population in Quebec [12], were chosen as the study sites: *l'Établissement de détention de Montréal* (EDM), *l'Établissement de détention de Rivière-des-Prairies* (EDRDP), and l'*Établissement de détention de St-Jérôme* (EDSJ). Both EDM and EDRDP are located in Montreal, the epicentre of the SARS-CoV-2 epidemic in Quebec, whereas EDSJ is located in the Laurentian region. As women represent approximately 10% of the incarcerated population in Quebec [13], we restricted our study population to men.

EDM is the largest provincial prison in Quebec, with a capacity of 1,400 individuals pre-pandemic [12]. During the second and third waves of the pandemic (Supplementary material, Table S1), EDM housed approximately 800 men [2]. EDRDP primarily houses individuals awaiting sentencing (on remand) and has a capacity of 541 [12]. During the second wave of the pandemic, EDRDP housed approximately 350 individuals [2]. EDSJ has a capacity of 587, but only housed 300 during the third and fourth waves of the pandemic [2,12]. There were two SARS-CoV-2 outbreaks per site during the study period (Supplementary material, Table S2); 268, 38, 113 incarcerated people and 135, 27, 63 correctional employees at EDM, EDRDP, and EDSJ, respectively, tested positive for SARS-CoV-2 as of September 15, 2021 – the last day of study recruitment.

**Participants**

We included individuals aged 18 years or older who were incarcerated for more than 24 hours and able to consent to study participation in either English or French. We excluded individuals who were both in isolation with active SARS-CoV-2 or under investigation for COVID-19 as a close contact of a diagnosed case as the research team was denied access to these individuals, and those who posed a security risk to the research team. Participants provided written informed consent and received an honorarium of $10 CAD for their study participation. This study was approved by the *McGill University Health Centre Research Ethics Board* (MUHC REB #2021–6888) and the *Direction régionale des services correctionnels du Québec* (#2020–12493).

The recruitment period spanned over nine months (January 19 to September 15, 2021) due to limited access to the study sites during COVID-19 prison outbreaks (Supplementary material, Table S2). Individuals were recruited across the three sites until 1,100 were consented. This sample size (n=1,087) was chosen to estimate a SARS-CoV-2 seroprevalence within a 2% margin of error (exact binomial formula) [19], assuming that seroprevalence would parallel at least that which was measured among Montreal blood donors after the second wave (i.e., 13%) [20]. The number of participants recruited from each site was proportional to the study site population.

## Data collection

Convenience sampling of individuals meeting the eligibility criteria was undertaken. Incarcerated individuals were approached in their cells by the research team, where the study was described in detail, and participants who agreed to participate were given self-administered questionnaires to complete in their cells or in the designated research space while awaiting serology testing. The questionnaire included questions on sociodemographic characteristics, COVID-19 clinical symptoms, risk factors and relevant exposures, general health, carceral conditions, and vaccination status. Individuals requiring assistance with reading and writing could request support from the research team.

SARS-CoV-2 serology testing was performed using the Roche Elecsys® anti-SARS-CoV-2 serology test. This test targets the SARS-CoV-2 nucleocapsid proteins, detecting immunoglobulin (Ig)G antibodies in human serum [21]. The serology test has a specificity of >99·8% and a sensitivity of 99·5% (14 days post-PCR confirmation) [21]. Given that Health Canada-approved mRNA COVID-19 vaccines induce spike protein-specific antibodies, the Roche Elecsys® anti-SARS-CoV-2 serology test was deliberately chosen as it does not cross-react with vaccine-induced antibodies, leading to false positive results among vaccinated participants. Samples were collected as whole blood and centrifuged within two hours. Samples were processed at *Sacré-Coeur Hospital* (Montreal) within 48 hours. Participants were given anonymized written memos of their test results by a research nurse within 72 hours of serology testing.

## Statistical analysis

The primary outcome measure was SARS-CoV-2 seropositivity, measured as a positive result to the anti-SARS-CoV-2 serology test. Independent variables were selected based on a literature review of factors associated with SARS-CoV-2 seroprevalence among incarcerated individuals and other vulnerable populations [22,23]. Summary statistics were calculated to describe the study sample: medians and interquartile ranges (IQR) for continuous variables and counts and proportions for categorical variables. Variables were grouped into the following categories: sociodemographic (age, ethnicity, education level, and housing status), clinical (medical comorbidities), and carceral characteristics (provincial prison, time spent incarcerated since March 2020, room type, meal consumption, prison employment, and timing of incarceration at screening relative to a prison outbreak). Time spent incarcerated, room type, meal consumption, and prison employment were measured by participant responses to the following questions, respectively: "*Since March 2020, how much time in total did you spend in a Quebec provincial prison*?" (little (<10%) vs. some (10–49%) vs. most (50–99%) vs. all (100%)), "*Since March 2020, have you shared your cell with another inmate?*" (yes vs. no), "*Since March 2020, who have you primarily had meals with?*" (alone vs. with cellmates vs. with sector), and "*Have you been working in a detention facility (e.g. food service, cleaning, inmate committee, etc.) since January 1, 2020*" (yes vs. no). March 2020 corresponded to the beginning of the first SARS-CoV-2 wave in Quebec [24]. Timing of incarceration at screening was measured based on dates of study participation and extrapolated to represent either pre- or post-prison outbreak.

Poisson regression models with robust standard errors were used to examine the effect of carceral exposures on SARS-CoV-2 seropositivity. Specifically, we used directed acyclic graphs (DAGs) [25] to depict known or plausible relationships between selected modifiable carceral exposures and the outcomes. These DAGs were used to identify confounders for inclusion in multivariable regression models (Supplementary material, Figure S1). Since the effect of an exposure on the outcome can be mediated [26], separate multivariable models were constructed for each carceral exposure of interest and their total effect on SARS-CoV-2 seropositivity estimated, resulting in five sets of adjustment variables (Supplementary material, Table S3). Crude and adjusted prevalence ratios (aPR) with 95% confidence intervals (95%CI) were calculated. Fixed effects for prisons were included to account for clustering of participants by correctional facilities.

Multiple imputation was performed to reduce bias attributable to missing observations, under the assumption of missing at random. A total of five imputed data sets were obtained and results from these regression models were combined using Rubin's rule. All analyses were performed using R statistical software (version 4.0.3) and the "geepack" library.

## Results

### Sample characteristics

A total of 2,170 incarcerated individuals across the three provincial prisons were invited to participate (n=1,181 at EDM, n=549 at EDRDP, and n=440 at EDSJ). Of these, 1,056 (49%) declined participation (Figure 1); more than half of whom were not interested in participating in research. An additional 14 participants were excluded, leaving 1,100 participants (n=600 at EDM, n=300 at EDRDP, and n=200 at EDSJ).

Overall, the median age was 37 years (Table 1). Approximately two-thirds (64%) of participants self-identified as White. The majority (71%) had a secondary school level education or less. Half reported a personal gross yearly income of less than $30,000 CAD and 26% reported unstable housing prior to incarceration. Less than half (42%) reported at least one COVID-19 related symptom since January 1, 2020. Approximately half of participants reported at least one chronic health condition. One-third spent most (≥50%) of their time incarcerated since March 2020. The majority were housed in shared cells and were unemployed during incarceration; two-thirds consumed their meals with cellmates or within their sector. The frequency distributions of race/ethnicity, SARS-CoV-2 serology test result, time spent incarcerated, room type, meal consumption, and timing of incarceration differed across correctional facilities.

**SARS-CoV-2 seropositivity**

A total of 246 (22%) participants tested positive to the anti-SARS-CoV-2 serology test: 164 (27%) at EDM, 44 (15%) at EDRDP, and 38 (19%) at EDSJ. Of these, 192 (78%) reported having at least one previous SARS-CoV-2 PCR test, with 122 (64%) testing positive and 70 (36%) testing negative. Among the 122, 83 (68%) reported testing only in detention; 41 of 79 (52%) with available incarceration information reported being permanently incarcerated since March 2020. Among the 854 participants who tested negative, 493 (58%) previously underwent SARS-CoV-2 PCR testing and a minority (2%) reported a prior positive test result. A total of 73 (30%) participants with positive serology test results reported no history of COVID-19 symptoms.

Risk factors for SARS-CoV-2 seropositivity identified in univariate analyses are presented in Table 2. In univariate analysis, Black or other visible minority, unstable housing, COVID-19 symptoms, incarceration at EDM, time spent in prison, employment during incarceration, and incarceration post-outbreak were associated with higher SARS-CoV-2 seroprevalence. In the multivariable models examining the causal effect of various carceral exposures on SARS-CoV-2 seropositivity (Table 3), seropositivity increased with time spent incarcerated ("*most time*": aPR 1.47, 95%CI 1.01–2.12; "*all time*": aPR 2.17, 95%CI 1.53–3.07), employment during incarceration (aPR 1.64, 95%CI 1.28–2.11), shared meal consumption during incarceration ("*with cellmates*": aPR 1.46, 95%CI 1.08–1.97; "*with sector*": aPR 1.34, 95%CI 1.03–1.74), and incarceration post-outbreak (aPR 2.32, 95%CI 1.69–3.18). The type of room occupied during incarceration (single cell vs. shared cell) was not associated with SARS-CoV-2 seropositivity.

**Discussion**

Our study offers the first description of SARS-CoV-2 seroprevalence in the Canadian incarcerated population, and to date, the largest seroprevalence study to be conducted in a correctional setting.

We observed a seroprevalence that was two-fold higher than non-vaccinated individuals in Montreal (13.75%) after the second SARS-CoV-2 wave in Quebec [20]. Although our study differs in sampling and population, our findings likely reflect both a high-risk carceral environment and a population that may be at heightened risk in the community through communal or unstable housing and occupational risk. Our study also found that several modifiable carceral risk factors, such as time spent in prison, employment and shared meal consumption during incarceration, and incarceration post-prison outbreak were associated with increased seropositivity. These findings have important implications on public health policies and are of utmost importance until COVID-19 vaccine uptake is high in Canadian provincial prisons.

While we observed a relatively high SARS-CoV-2 seroprevalence among incarcerated individuals, it varied between prisons, ranging from 15% to 27%. This variability likely reflects several factors including the type of incarcerated population in each prison. We found that the prison (EDRDP) which primarily houses individuals awaiting sentencing (i.e., on remand) had a SARS-CoV-2 seroprevalence (15%) that paralleled the surrounding community, reflecting the high turnover of on remand individuals, while the seroprevalence at EDM and EDSJ, which house both shorter- and longer-sentenced individuals, was higher, reflecting the additional risk of incarceration. Differences in seroprevalence were also likely due to the timing of recruitment with respect to prison-based and provincial SARS-CoV-2 outbreaks, as another study has shown [17]. Finally, there are important structural differences (size and spatial organization) between prisons, and a variable number of correctional employees at each prison, further contributing to differences in overall SARS-CoV-2 seroprevalence.

We found a time-dependent association between duration of incarceration and seropositivity, suggesting that decarceration is an important strategy in preventing SARS-CoV-2 outbreaks in correctional settings. Decarceration consists of the large-scale release of people who pose minimal risk to public safety, the increased use of home confinement, and the non-carceral management of people arrested for minor offenses [27]. Decarceration, when paired with basic preventative measures, is effective in reducing prison-based SARS-CoV-2 transmission [28-29] and leads to population-level public health benefits [29]. Such benefits, however, depend on appropriate community re-integration assistance at the time of release. Otherwise, individuals may be liberated only to find temporary housing in shelters or communal spaces, environments that are equally prone to SARS-CoV-2 outbreaks [23]. This underscores the use of best practices for implementing decarceration as a mitigation strategy by ensuring that conditions that support safe and successful re-entry of those decarcerated are met [30]. Decarceration also entails that unnecessary prison admissions are reduced. Prosecution for misdemeanors and other minor crimes such as drug possession could be entirely deferred to minimize the overall prison population. These efforts require collaboration from stakeholders across several disciplines and could have a dramatic effect on preventing SARS-CoV-2 outbreaks in prisons.

Given the very high transmission potential in congregate settings, layering multiple preventative interventions is important. Enhanced occupational safety measures for those who are employed during incarceration are needed [31], and the consideration of strict individual meal consumption when there is ongoing risk for SARS-CoV-2 transmission. Furthermore, while we did not find an increased risk of seropositivity with shared cells, studies have shown that “dormitory housing” is a risk factor for acute SARS-CoV-2 infection [22,31,32] and that converting cells into single occupancy may reduce SARS-CoV-2 transmission in correctional settings [33]. In addition, the relatively high (30%) prevalence of asymptomatic infection undermines prison-based surveillance measures that only test symptomatic individuals and their contacts. A shift towards a broad-based testing approach, including mandatory testing on admission, should be considered going forward [6,32].

Our study also demonstrated that the capacity to implement infection prevention and control measures in correctional settings is limited, underscoring that vaccination is a necessary component of the preventative armamentarium [8]. In fact, a recent study found that the effectiveness of mRNA vaccines in U.S. prisons was equivalent to randomized trials and observational studies [34], highlighting the importance of accelerated, large-scale COVID-19 vaccine roll-out in correctional settings. That said, studies have shown that after achieving high vaccine coverage in prisons, ongoing infection prevention and control measures are necessary to prevent future outbreaks in the viral variant era [35], suggesting that a multimodal approach will likely be required going forward.

There are limitations to our study. First, our study sites were not randomly selected, but were chosen based on practical considerations including proximity to laboratory facilities in Montreal. This restricted our study population to adult men incarcerated in three of 16 provincial prisons, albeit representing almost half of Quebec's incarcerated male provincial population. Due to security constraints, we used convenience sampling for recruitment. Among individuals approached for participation, almost half (49%) declined study participation. We were not able to collect demographic information among these individuals, which could have informed whether our study population deviates from the overall prison populations, as well as the possibility for selection bias. Furthermore, all three study sites had multiple outbreaks during study recruitment. Our results may thus not be generalizable to other facilities which did not experience a SARS-CoV-2 outbreak, or to those which primarily house women. Second, we likely underestimated the SARS-CoV-2 seroprevalence for several reasons. We excluded individuals with suspected or confirmed COVID-19 infection as they were in isolation with restricted access. These individuals; however, became eligible to participate following the completion of their isolation. Further, as recruitment occurred longitudinally and not simultaneously at each of the three sites, it is possible that infection-induced antibodies waned over time, increasing the possibility for seroreversion. Third, the cross-sectional study design precludes our ability to determine when and where participants acquired SARS-CoV-2 infection, be it in prison or the community. While our questionnaire inquired where previous testing occurred and about test results, various unmeasured variables related to infection were not collected, thereby impacting our inferences. Fourth, information related to carceral conditions, potential exposures, and risk factors were collected from study questionnaires, which may have introduced response biases such as acquiescence, social desirability, and dissent biases. However, the impact of these biases was likely limited with the use of self-administered questionnaires, and the inclusion of non-correctional nurses in the consent process. Fifth, several steps were taken to mitigate biases. We used DAGs to identify confounders to control for while obtaining total effects, and multiple imputation was used to address missing data. Finally, while several studies have attempted to estimate the risk of infection in correctional settings, the majority estimated prevalence of active infection based on PCR testing [6,32,36-38] – testing that was often symptom-based or reactive post-outbreak – underscoring the contribution of our study to the dearth of seroprevalence data among this vulnerable population. More importantly, very few studies explored carceral risk factors associated with SARS-CoV-2 seropositivity [22,31,32], highlighting the important role of our study to policy makers and public health experts.

In conclusion, we found a high prevalence of exposure to SARS-CoV-2 among men in the provincial prison system in Quebec. There were several modifiable carceral factors that were associated with an increased risk of SARS-CoV-2 seropositivity, highlighting the need for a multimodal approach in preventing future outbreaks. Strategies that seek to mitigate prison-based outbreaks should consist of decarceration, individual meal consumption and single cell occupancy, enhanced occupational safety measures and infection prevention and control measures including vaccination during incarceration.

**NOTES**

**Funding**

This work was supported by the *Public Health Agency of Canada* through the Sero-Surveillance and Research (COVID-19 Immunity Task Force Initiative) Program (#2021-HQ-000103). The funders had no role in the study design, data collection, data analysis, data interpretation, or writing of the report. NK is supported by a career award from the Fonds de Recherche Québec – *Santé (FRQ-S; Junior 1*). MMG is supported by a Canada Research Chair (Tier 2) in *Population Health Modeling*.

**Acknowledgements**

The authors would like to thank all the participants of the seroprevalence study, as well as the Directors of Professional Services at the three provincial prisons who granted access to the research team during the COVID-19 pandemic. The opinions and conclusions presented do not necessarily represent those of the *Ministère de la sécurité publique du Québec*. The authors would also like to thank those in the respective Departments of Finance for compensating participants in a timely manner, and to the many correctional agents who ensured the safety of the research team. Finally, the authors would like to thank the lab technicians at Sacré-Coeur Hospital for ensuring the rapid turn-around of test results.

**Conflict of interest**

CD, AH, SC, JS, HP, LDB, and SP declare no competing interests. NK reports research funding from Gilead Sciences, McGill Interdisciplinary Initiative in Infection and Immunity, Canadian Institutes of Health Research, and Canadian Network on Hepatitis C, advisory fees from Gilead Sciences, ViiV Healthcare, Merck and Abbvie, and speaker fees from Gilead

Sciences, Abbvie and Merck, all outside of the submitted work. MMG reports an investigator-sponsored research grant from Gilead Sciences Inc. MMG reports contractual arrangements with the World Health Organization, the *Institut national de santé publique du Québec*, and the *Institut d'excellence en santé et services sociaux du Québec*, all outside of the submitted work. MPC reports grants from the McGill Interdisciplinary Initiative in Infection and Immunity and from the Canadian Institutes of Health Research. MPC reports personal fees from GEn1E Lifesciences and form nplex biosciences, both outside the submitted work. MPC is the co-founder of Kanvas Biosciences, Inc. and owns equity in the company. MPC has a pending patent for Methods for detecting tissue damage, graft versus host disease, and infections using cell-free DNA profiling pending (Cornell Reference No: 9401-01-US

Pending), and a pending patent for Methods for assessing the severity and progression of SARS-CoV-2 infections using cell-free DNA (Cornell Reference No: 9561-01-US

Pending). JC has research funding from ViiV Healthcare for an investigator initiated study and Gilead Sciences for clinical trials, and reports remuneration for advisory work (ViiV Healthcare, Gilead Sciences and Merck Canada), outside the submitted work.

**Table 1: Baseline characteristics among adult men in three provincial prisons in Quebec, Canada (2021).**

| | EDM | EDRDP | EDSJ | Total |
|---|---|---|---|---|
| | N = 600 (55%) | N = 300 (27%) | N = 200 (18%) | N=1,100 |
| **Sociodemographic characteristics** | | | | |
| **Age (mean [SD]), years** | 39.0 [12.3] | 38.2 [12.6] | 38.5 [12.8] | 38.7 [12.5] |
| **Age category – n (%)** | | | | |
| 18–29 years | 149 (25%) | 92 (31%) | 52 (27%) | 293 (26%) |
| 30–39 years | 192 (32%) | 76 (25%) | 61 (30%) | 329 (30%) |
| 40–49 years | 136 (23%) | 66 (22%) | 49 (24%) | 251 (23%) |
| ≥50 years | 123 (20%) | 66 (22%) | 38 (19%) | 227 (21%) |
| **Race/ethnicity – n (%)** | | | | |
| White, non-Hispanic | 392 (65%) | 178 (59%) | 136 (68%) | 706 (64%) |
| Black, non-Hispanic | 77 (13%) | 45 (15%) | 4 (2%) | 126 (12%) |
| Indigenous | 57 (9%) | 26 (9%) | 52 (26%) | 135 (12%) |
| Other visible minority[a] | 64 (11%) | 44 (15%) | 4 (2%) | 112 (10%) |
| *Missing data* | *10 (2%)* | *7 (2%)* | *4 (2%)* | *21 (2%)* |
| **Education level – n (%)** | | | | |

| | | | | |
|---|---|---|---|---|
| Less than secondary | 211 (35%) | 107 (35%) | 85 (43%) | 403 (37%) |
| Secondary | 216 (36%) | 104 (35%) | 55 (27%) | 375 (34%) |
| Post-secondary | 172 (29%) | 87 (29%) | 50 (25%) | 309 (28%) |
| *Missing data* | *1 (0.2%)* | *2 (1%)* | *10 (5%)* | *13 (1%)* |
| **Personal gross yearly income[b] (CAD) – n (%)** | | | | |
| $0 or no income | 75 (13%) | 51 (17%) | 16 (8%) | 142 (13%) |
| $1–$29,999 | 228 (38%) | 122 (41%) | 76 (38%) | 426 (39%) |
| $30,000–$59,999 | 102 (17%) | 52 (17%) | 38 (19%) | 192 (17%) |
| ≥$60,000 | 73 (12%) | 30 (10%) | 23 (12%) | 126 (11%) |
| *Missing data* | *122 (20%)* | *45 (15%)* | *47 (23%)* | *214 (20%)* |
| **Housing status[c] – n (%)** | | | | |
| Unstable | 155 (26%) | 92 (30%) | 37 (18%) | 284 (26%) |
| Stable | 415 (69%) | 203 (68%) | 151 (76%) | 769 (70%) |
| *Missing data* | *30 (5%)* | *5 (2%)* | *12 (6%)* | *47 (4%)* |
| **Clinical characteristics** | | | | |
| **COVID-19 symptoms[d] – n (%)** | | | | |
| No | 328 (55%) | 160 (53%) | 116 (58%) | 604 (55%) |

| | | | | |
|---|---|---|---|---|
| Yes | 257 (43%) | 134 (45%) | 76 (38%) | 467 (42%) |
| *Missing data* | *15 (2%)* | *6 (2%)* | *8 (4%)* | *29 (3%)* |
| **Medical comorbidities[e] – n (%)** | | | | |
| None | 299 (50%) | 152 (51%) | 103 (52%) | 554 (50%) |
| One | 187 (31%) | 98 (33%) | 68 (34%) | 353 (32%) |
| $\geq$ 2 | 80 (13%) | 40 (13%) | 22 (11%) | 142 (13%) |
| *Missing data* | *34 (6%)* | *10 (3%)* | *7 (3%)* | *51 (5%)* |
| **SARS-CoV-2 serology test result – n (%)** | | | | |
| Negative | 436 (73%) | 256 (85%) | 162 (81%) | 854 (78%) |
| Positive | 164 (27%) | 44 (15%) | 38 (19%) | 246 (22%) |
| **Carceral characteristics** | | | | |
| **Time spent incarcerated since March 2020 – n (%)** | | | | |
| Little (<10%)[f] | 168 (28%) | 86 (29%) | 35 (17%) | 289 (26%) |
| Some (10–49%) | 162 (27%) | 81 (27%) | 88 (44%) | 331 (30%) |
| Most (50–99%) | 103 (17%) | 61 (20%) | 23 (12%) | 187 (17%) |
| All (100%) | 110 (18%) | 46 (15%) | 20 (10%) | 176 (16%) |

| | | | | |
|---|---|---|---|---|
| *Missing data* | *57 (10%)* | *26 (9%)* | *34 (17%)* | *117 (11%)* |
| **Room type – n (%)** | | | | |
| Single cell | 128 (21%) | 30 (10%) | 15 (7%) | 173 (16%) |
| Shared cell | 460 (77%) | 266 (89%) | 181 (91%) | 907 (82%) |
| *Missing data* | *12 (2%)* | *4 (1%)* | *4 (2%)* | *20 (2%)* |
| **Employment during incarceration – n (%)** | | | | |
| No | 484 (81%) | 242 (81%) | 156 (78%) | 882 (80%) |
| Yes | 72 (12%) | 38 (12%) | 32 (16%) | 142 (13%) |
| *Missing data* | *44 (7%)* | *20 (7%)* | *12 (6%)* | *76 (7%)* |
| **Meal consumption – n (%)** | | | | |
| Alone | 190 (32%) | 81 (27%) | 36 (18%) | 307 (28%) |
| Cellmates | 143 (24%) | 78 (26%) | 19 (10%) | 240 (22%) |
| Sector | 214 (35%) | 132 (44%) | 135 (67%) | 481 (44%) |
| *Missing data* | *53 (9%)* | *9 (3%)* | *10 (5%)* | *72 (6%)* |
| **Timing of incarceration at screening – n (%)** | | | | |
| Pre-outbreak | 130 (22%) | 0 (0%) | 106 (53%) | 236 (22%) |
| Post-outbreak | 470 (78%) | 300 (100%) | 94 (47%) | 864 (78%) |

EDM: Établissement de détention de Montréal; EDRDP: Établissement de détention de Rivière-des-Prairies; EDSJ: Établissement de détention de Saint-Jérôme; SD: Standard deviation.

[a]Other visible minority includes Hispanic, Asian, and Arab.

[b]Personal gross yearly income refers to total annual income (CAD) from all paid work and all other sources before taxes and other deductions in the year prior to incarceration.

[c]Stable housing refers to living in an apartment, condo or house; unstable housing refers to living in a shelter, group home, hotel or having no fixed address.

[d]COVID-19 symptoms include fever, chills, headache, sore throat, new or worsening cough, stuffy nose/congestion, difficulty breathing/shortness of breath, loss of smell or taste, fatigue, weakness, confusion, diarrhea, muscle pain, vomiting and nausea.

[e]Medical comorbidities include hypertension, diabetes, obesity (based on body mass index), asthma, chronic lung disease, chronic heart disease, chronic kidney disease, liver disease, cancer, chronic blood disorder, chronic neurological disorder, immunocompromised (HIV), immunocompromised (Other).

[f]Reported spending less than 4 weeks incarcerated since March 2020.

**Table 2: Unadjusted associations between the risk factors of interest and anti-SARS-CoV-2 seropositivity among adult men in three provincial prisons in Quebec, Canada (2021).**

| | **Prevalence ratio** | **95%CI** |
|---|---|---|
| **Sociodemographic characteristics** | | |
| **Age category** | | |
| 18–29 years | *Reference* | *Reference* |
| 30–39 years | 0.84 | 0.63–1.13 |
| 40–49 years | 0.84 | 0.61–1.15 |
| ≥50 years | 1.04 | 0.77–1.40 |
| **Race/ethnicity** | | |
| White, non-Hispanic | *Reference* | *Reference* |
| Black, non-Hispanic | **1.55** | **1.15–2.08** |
| Indigenous | 0.96 | 0.66–1.39 |
| Other visible minority[a] | **1.45** | **1.05–1.99** |
| **Education level** | | |
| Less than secondary | *Reference* | |
| Secondary | 1.10 | 0.85–1.43 |

| | | |
|---|---|---|
| Post-secondary | 1.07 | 0.81–1.41 |
| **Housing status**[b] | | |
| Stable | *Reference* | *Reference* |
| Unstable | **1.51** | **1.21–1.89** |
| **Clinical characteristics** | | |
| **COVID-19 symptoms**[c] | | |
| No | *Reference* | *Reference* |
| Yes | **2.93** | **2.30–3.74** |
| **Medical comorbidities**[d] | | |
| None | *Reference* | *Reference* |
| 1 | 0.98 | 0.76–1.25 |
| ≥2 | 1.15 | 0.84–1.57 |
| **Carceral characteristics** | | |
| **Provincial prison** | | |
| EDRDP | *Reference* | *Reference* |
| EDM | **1.86** | **1.38–2.52** |
| EDSJ | 1.30 | 0.87–1.92 |
| **Time spent incarcerated since March** | | |

| | | |
|---|---|---|
| **2020** | | |
| Little (<10%)[e] | *Reference* | *Reference* |
| Some (10–49%) | 1.38 | 0.99–1.92 |
| Most (50–99%) | **1.59** | **1.11–2.28** |
| All (100%) | **2.73** | **1.99–3.74** |
| **Room type** | | |
| Single cells | *Reference* | *Reference* |
| Shared cells | 0.92 | 0.69–1.22 |
| **Employment during incarceration** | | |
| No | *Reference* | *Reference* |
| Yes | **1·61** | **1.24–2.08** |
| **Meal consumption** | | |
| Alone | *Reference* | *Reference* |
| Cellmates | 1.26 | 0.93–1.73 |
| Sector | 1.28 | 0.97–1.67 |
| **Timing of incarceration at screening** | | |
| Pre-prison outbreak | *Reference* | *Reference* |
| Post-prison outbreak | **1.76** | **1.26–2.47** |

CI: Confidence interval; EDRDP: Établissement de détention de Rivière-des-Prairies; EDM: Établissement de détention de Montréal; EDSJ: Établissement de détention de Saint-Jérôme.

[a]Other visible minority includes Hispanic, Asian, and Arab.

[b]Stable housing refers to living in an apartment, condo or house; unstable housing refers to living in a shelter, group home, hotel or having no fixed address.

[c]COVID-19 symptoms include fever, chills, headache, sore throat, new or worsening cough, stuffy nose/congestion, difficulty breathing/shortness of breath, loss of smell or taste, fatigue, weakness, confusion, diarrhea, muscle pain, vomiting and nausea.

[d]Medical comorbidities include hypertension, diabetes, obesity (based on body mass index), asthma, chronic lung disease, chronic heart disease, chronic kidney disease, liver disease, cancer, chronic blood disorder, chronic neurological disorder, immunocompromised (HIV), immunocompromised (other).

[e]Reported spending less than 4 weeks incarcerated since March 2020.

**Table 3: Adjusted associations between carceral exposures of interest and anti-SARS-CoV-2 seropositivity among adult men in three provincial prisons in Quebec, Canada (2021).**

| **Models** | **Carceral exposures** | **aPR** | **95%CI** |
|---|---|---|---|
| Model 1[a] | **Time spent incarcerated since March 2020** | | |
| | Little (<10%) | *Reference* | *Reference* |
| | Some (10–49%) | 1.32 | 0.95–1.85 |
| | Most (50–99%) | **1.47** | **1.01–2.12** |
| | All (100%) | **2.17** | **1.53–3.07** |
| Model 2[b] | **Room type** | | |
| | Single cells | *Reference* | *Reference* |
| | Shared cells | 1.03 | 0.77–1.36 |
| Model 3[c] | **Employment during incarceration** | | |
| | No | *Reference* | *Reference* |
| | Yes | **1.64** | **1.28–2.11** |
| Model 4[d] | **Meal consumption** | | |
| | Alone | *Reference* | *Reference* |
| | Cellmates | **1.46** | **1.08–1.97** |

| | | | |
|---|---|---|---|
| | Sector | **1.34** | **1.03–1.74** |
| Model 5[e] | **Timing of incarceration at screening** | | |
| | Pre-prison outbreak | *Reference* | *Reference* |
| | Post-prison outbreak | **2.32** | **1.69–3.18** |

aPR: Adjusted prevalence ratio; CI: Confidence interval.

[a]Adjusted for age, race/ethnicity, education, housing status, provincial prison, and employment status during incarceration.

[b]Adjusted for medical comorbidities, provincial prison, employment status during incarceration, and prison outbreak.

[c]Adjusted for age, education, medical comorbidities and provincial prison.

[d]Adjusted for COVID-19 symptoms, provincial prison, room type, employment status during incarceration, and prison outbreak.

[e]Adjusted for age, medical comorbidities, provincial prison, room type, meal consumption, and employment status during incarceration.

**Figure 1: Sample selection flow chart of study participants at the three provincial prisons in Quebec, Canada (2021).**

## Supplementary Material

ciac031_suppl_Supplementary_Material

