# Seroprevalence and Risk Factors for Severe Acute Respiratory Syndrome Coronavirus 2 Among Incarcerated Adult Men in Quebec, Canada, 2021

**DOI:** 10.1093/cid/ciac031

**Published:** 2022-01-17

**Authors:** Nadine Kronfli, Camille Dussault, Mathieu Maheu-Giroux, Alexandros Halavrezos, Sylvie Chalifoux, Jessica Sherman, Hyejin Park, Lina Del Balso, Matthew P Cheng, Sébastien Poulin, Joseph Cox

**Affiliations:** Centre for Outcomes Research and Evaluation, Research Institute of the McGill University Health Centre, Montréal, Québec, Canada; Department of Medicine, Division of Infectious Diseases and Chronic Viral Illness Service, McGill University Health Centre, Montréal, Québec, Canada; Centre for Outcomes Research and Evaluation, Research Institute of the McGill University Health Centre, Montréal, Québec, Canada; Department of Epidemiology and Biostatistics, School of Population and Global Health, Faculty of Medicine and Health Sciences, McGill University, Montréal, Québec, Canada; Centre for Outcomes Research and Evaluation, Research Institute of the McGill University Health Centre, Montréal, Québec, Canada; Centre for Outcomes Research and Evaluation, Research Institute of the McGill University Health Centre, Montréal, Québec, Canada; Centre for Outcomes Research and Evaluation, Research Institute of the McGill University Health Centre, Montréal, Québec, Canada; Centre for Outcomes Research and Evaluation, Research Institute of the McGill University Health Centre, Montréal, Québec, Canada; Centre for Outcomes Research and Evaluation, Research Institute of the McGill University Health Centre, Montréal, Québec, Canada; Department of Medicine, Divisions of Infectious Diseases and Medical Microbiology, McGill University Health Centre, Montréal, Québec, Canada; Centre intégré de santé et de services sociaux des Laurentides, Saint-Jérôme, Québec, Canada; Centre for Outcomes Research and Evaluation, Research Institute of the McGill University Health Centre, Montréal, Québec, Canada; Department of Medicine, Division of Infectious Diseases and Chronic Viral Illness Service, McGill University Health Centre, Montréal, Québec, Canada; Department of Epidemiology and Biostatistics, School of Population and Global Health, Faculty of Medicine and Health Sciences, McGill University, Montréal, Québec, Canada

**Keywords:** SARS-CoV-2, seroprevalence, antibody, incarceration, prison

## Abstract

**Background:**

People in prison are at increased risk of severe acute respiratory syndrome coronavirus 2 (SARS-CoV-2) infection. We examined the seroprevalence of SARS-CoV-2 and associated carceral risk factors among incarcerated adult men in Quebec, Canada.

**Methods:**

We conducted a cross-sectional seroprevalence study in 2021 across 3 provincial prisons, representing 45% of Quebec’s incarcerated male provincial population. The primary outcome was SARS-CoV-2 antibody seropositivity (Roche Elecsys serology test). Participants completed self-administered questionnaires on sociodemographic, clinical, and carceral characteristics. The association of carceral variables with SARS-CoV-2 seropositivity was examined using Poisson regression models with robust standard errors. Crude and adjusted prevalence ratios (aPR) with 95% confidence intervals (95% CIs) were calculated.

**Results:**

Between 19 January 2021 and 15 September 2021, 246 of 1100 (22%) recruited individuals tested positive across 3 prisons (range, 15%–27%). Seropositivity increased with time spent in prison since March 2020 (aPR, 2.17; 95% CI, 1.53–3.07 for “all” vs “little time”), employment during incarceration (aPR, 1.64; 95% CI, 1.28–2.11 vs not), shared meal consumption during incarceration (“with cellmates”: aPR, 1.46; 95% CI, 1.08–1.97 vs “alone”; “with sector”: aPR, 1.34; 95% CI, 1.03–1.74 vs “alone”), and incarceration post-prison outbreak (aPR, 2.32; 95% CI, 1.69–3.18 vs “pre-outbreak”).

**Conclusions:**

The seroprevalence of SARS-CoV-2 among incarcerated individuals was high and varied among prisons. Several carceral factors were associated with seropositivity, underscoring the importance of decarceration and occupational safety measures, individual meal consumption, and enhanced infection prevention and control measures including vaccination during incarceration.

## Teaser

Severe acute respiratory syndrome coronavirus 2 (SARS-CoV-2) seroprevalence was high among incarcerated adult men in Quebec, Canada. Several carceral factors were associated with seropositivity, underscoring decarceration and infection prevention and control measures such as vaccination in preventing future SARS-CoV-2 outbreaks.

Canadian correctional settings have witnessed several large outbreaks of novel severe acute respiratory syndrome coronavirus 2 (SARS-CoV-2) since the start of the pandemic [1, 2]. Overcrowding, poor ventilation, unsanitary conditions, limited testing, and challenges in accessing and implementing effective infection prevention and control measures [3–7] have accelerated transmission of SARS-CoV-2 among those living in correctional facilities, resulting in levels of transmission that are several-fold higher than in most surrounding communities [3, 6, 8]. An aging and comorbid Canadian carceral population [9] and the disproportionate incarceration of people experiencing social and health inequities [10, 11] underscore the importance of implementing prison-based preventative measures to mitigate future outbreaks in correctional settings.

In Canada, an estimated 38 000 people are incarcerated each day—14 000 in federal custody and 24 000 in provincial/territorial custody [9]. Since the start of the coronavirus disease 2019 (COVID-19) pandemic, the average daily incarcerated provincial population in Quebec (approximately 4500 individuals) was reduced by 20%, a deliberate preventative measure to reduce prison crowding through decreased justice system activities (eg, postponed trials, fewer arrests), lower incarceration rates, and the early release of low-risk individuals [2, 12, 13]. Furthermore, the Canadian National Advisory Committee on Immunization prioritized “resident and staff of congregate settings” for early COVID-19 vaccination in December 2020 [14]. However, COVID-19 vaccine rollout for people incarcerated in provincial prisons, including Quebec, has trailed the federal response by Correctional Service Canada [2, 15, 16]. With the delta variant, maintaining prison-based preventative measures until high vaccine coverage is achieved remains crucial, particularly with high prison-based transmission [7].

While more than 750 people incarcerated in Quebec’s provincial prisons have tested positive for SARS-CoV-2 since March 2020 [2], the start of the COVID-19 pandemic in Quebec, this likely underestimates the true extent of SARS-CoV-2 exposure in provincial prisons. Several reasons likely contribute to this including decreased disclosure of symptoms due to the mandatory quarantine or isolation of those who test SARS-CoV-2 polymerase chain reaction (PCR) positive, a focus on symptom-based testing [6], logistical difficulties of SARS-CoV-2 testing in prison settings, and the high turnover of those incarcerated in provincial prisons [9]. To reduce SARS-CoV-2 transmission, a mandatory 14-day isolation on admission, the provision of masks, and the cessation of interfacility transfers were introduced. Seroprevalence studies have been conducted in correctional settings in low- and middle-income countries [17, 18], where limited mitigation strategies likely contribute to higher SARS-CoV-2 seroprevalence. Therefore, we examined the seroprevalence of SARS-CoV-2 antibodies among people incarcerated in Quebec provincial prisons and determined the effects of carceral exposures on SARS-CoV-2 seropositivity.

## METHODS

### Study Design and Setting

We conducted an observational, cross-sectional study in 3 correctional facilities under the responsibility of the Ministère de la sécurité publique du Québec (MSP). The MSP oversees provincial corrections, where adult individuals serve sentences of less than 2 years [9]. Three large provincial correctional facilities, representing 45% of the incarcerated male provincial population in Quebec [12], were chosen as the study sites: l’Établissement de détention de Montréal (EDM), l’Établissement de détention de Rivière-des-Prairies (EDRDP), and l’Établissement de détention de St-Jérôme (EDSJ). Both EDM and EDRDP are located in Montreal, the epicenter of the SARS-CoV-2 epidemic in Quebec, whereas EDSJ is located in the Laurentian region. As women represent approximately 10% of the incarcerated population in Quebec [13], we restricted our study population to men.

EDM is the largest provincial prison in Quebec, with a capacity of 1400 individuals pre-pandemic [12]. During the second and third waves of the pandemic (Supplementary Table 1), EDM housed approximately 800 men [2]. EDRDP primarily houses individuals awaiting sentencing (on remand) and has a capacity of 541 [12]. During the second wave of the pandemic, EDRDP housed approximately 350 individuals [2]. EDSJ has a capacity of 587 but only housed 300 during the third and fourth waves of the pandemic [2, 12]. There were 2 SARS-CoV-2 outbreaks per site during the study period (Supplementary  Table 2); 268, 38, and 113 incarcerated people and 135, 27, and 63 correctional employees at EDM, EDRDP, and EDSJ, respectively, had tested positive for SARS-CoV-2 as of 15 September 2021, the last day of study recruitment.

### Participants

We included individuals aged ≥18 years who were incarcerated for more than 24 hours and able to consent to study participation in either English or French. We excluded individuals who were both in isolation with active SARS-CoV-2 or under investigation for COVID-19 as a close contact of a diagnosed case as the research team was denied access to these individuals and those who posed a security risk to the research team. Participants provided written informed consent and received an honorarium of $7.5 USD for their study participation. The McGill University Health Centre Research Ethics Board and the Direction régionale des services correctionnels du Québec approved the study.

The recruitment period spanned 9 months (19 January 2021 to 15 September 2021) due to limited access to the study sites during COVID-19 prison outbreaks (Supplementary Table 2). Individuals were recruited across the 3 sites until 1100 were consented. This sample size (n = 1087) was chosen to estimate a SARS-CoV-2 seroprevalence within a 2% margin of error (exact binomial formula) [19], assuming that seroprevalence would parallel at least that which was measured among Montreal blood donors after the second wave (ie, 13%) [20]. The number of participants recruited from each site was proportional to the study site population.

### Data Collection

Convenience sampling of individuals who met the eligibility criteria was undertaken. Incarcerated individuals were approached in their cells by the research team, where the study was described in detail, and participants who agreed to participate were given self-administered questionnaires to complete in their cells or in the designated research space while awaiting serology testing. The questionnaire included questions on sociodemographic characteristics, COVID-19 clinical symptoms, risk factors and relevant exposures, general health, carceral conditions, and vaccination status. Individuals who required assistance with reading and writing could request support from the research team.

SARS-CoV-2 serology testing was performed using the Roche Elecsys anti–SARS-CoV-2 serology test. This test targets the SARS-CoV-2 nucleocapsid proteins, detecting immunoglobulin G antibodies in human serum [21]. The serology test has a specificity of >99.8% and a sensitivity of 99.5% (14 days post-PCR confirmation) [21]. Given that Health Canada–approved mRNA COVID-19 vaccines induce spike protein–specific antibodies, the Roche Elecsys anti–SARS-CoV-2 serology test was deliberately chosen as it does not cross-react with vaccine-induced antibodies, leading to false-positive results among vaccinated participants. Samples were collected as whole blood and centrifuged within 2 hours. Samples were processed at Sacré-Coeur Hospital (Montreal) within 48 hours. Participants were given anonymized written memos of their test results by a research nurse within 72 hours of serology testing.

### Statistical Analyses

The primary outcome measure was SARS-CoV-2 seropositivity, measured as a positive result to the anti–SARS-CoV-2 serology test. Independent variables were selected based on a literature review of factors associated with SARS-CoV-2 seroprevalence among incarcerated individuals and other vulnerable populations [22, 23]. Summary statistics were calculated to describe the study sample: medians and interquartile ranges for continuous variables and counts and proportions for categorical variables. Variables were grouped into the following categories: sociodemographic (age, ethnicity, education level, and housing status), clinical (medical comorbidities), and carceral characteristics (provincial prison, time spent incarcerated since March 2020, room type, meal consumption, prison employment, and timing of incarceration at screening relative to a prison outbreak). Time spent incarcerated, room type, meal consumption, and prison employment were measured by participant responses to the following questions, respectively: “Since March 2020, how much time in total did you spend in a Quebec provincial prison?” (little [<10%] vs some [10%–49%] vs most [50%–99%] vs all [100%]), “Since March 2020, have you shared your cell with another inmate?” (yes vs no), “Since March 2020, who have you primarily had meals with?” (alone vs with cellmates vs with sector), and “Have you been working in a detention facility (eg, food service, cleaning, inmate committee) since January 1, 2020” (yes vs no). March 2020 corresponded to the beginning of the first SARS-CoV-2 wave in Quebec [24]. Timing of incarceration at screening was measured based on dates of study participation and extrapolated to represent either pre- or post-prison outbreak.

Poisson regression models with robust standard errors were used to examine the effect of carceral exposures on SARS-CoV-2 seropositivity. Specifically, we used directed acyclic graphs (DAGs) [25] to depict known or plausible relationships between selected modifiable carceral exposures and the outcomes. These DAGs were used to identify confounders for inclusion in multivariable regression models (Supplementary Figure 1). Since the effect of an exposure on the outcome can be mediated [26], separate multivariable models were constructed for each carceral exposure of interest and their total effect on SARS-CoV-2 seropositivity estimated, resulting in 5 sets of adjustment variables (Supplementary Table 3). Crude and adjusted prevalence ratios (aPRs) with 95% confidence intervals (95% CIs) were calculated. Fixed effects for prisons were included to account for clustering of participants by correctional facilities.

Multiple imputation was performed to reduce bias attributable to missing observations, under the assumption of missing at random. A total of 5 imputed datasets were obtained, and results from these regression models were combined using Rubin’s rule. All analyses were performed using R statistical software (version 4.0.3) and the “geepack” library.

## RESULTS

### Sample Characteristics

**Figure 1.**
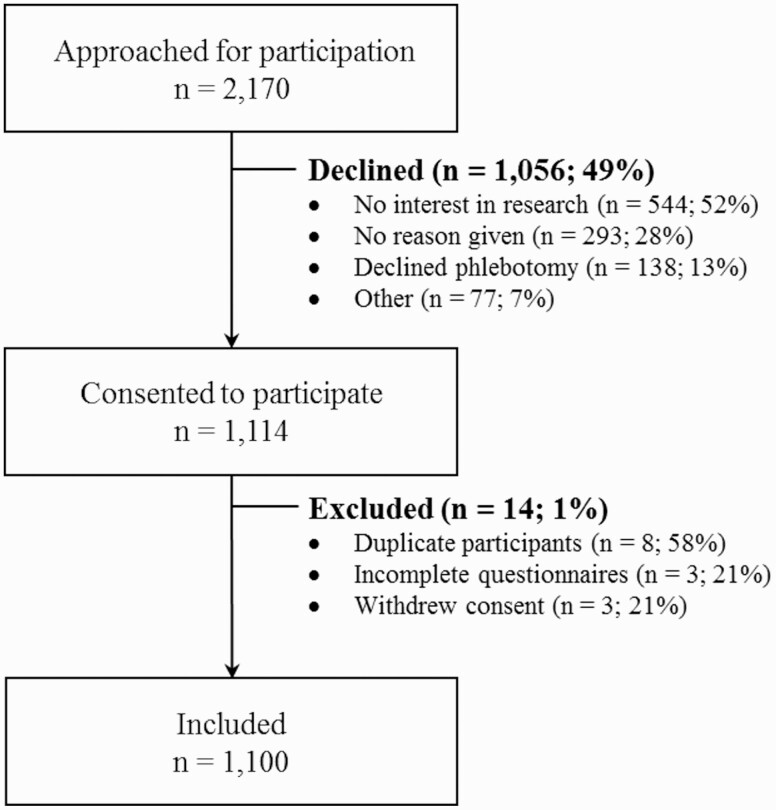
Sample selection flow chart of study participants at the 3 provincial prisons in Quebec, Canada, 2021.

A total of 2170 incarcerated individuals across the 3 provincial prisons were invited to participate (n = 1181 at EDM, n = 549 at EDRDP, and n = 440 at EDSJ). Of these, 1056 (49%) declined participation (Figure 1); more than half of whom were not interested in participating in research. An additional 14 participants were excluded, leaving 1100 participants (n = 600 at EDM, n = 300 at EDRDP, and n = 200 at EDSJ).

Overall, the median age was 37 years (Table 1). Approximately two-thirds (64%) of participants self-identified as White. The majority (71%) had a secondary school level education or less. Half reported a personal gross yearly income of less than $26,000 USD, and 26% reported unstable housing prior to incarceration. Less than half (42%) reported at least 1 COVID-19–related symptom since 1 January 2020. Approximately half of participants reported at least 1 chronic health condition. One-third had spent most (≥50%) of their time incarcerated since March 2020. The majority were housed in shared cells and were unemployed during incarceration; two-thirds consumed their meals with cellmates or within their sector. The frequency distributions of race/ethnicity, SARS-CoV-2 serology test result, time spent incarcerated, room type, meal consumption, and timing of incarceration differed across correctional facilities.

**Table 1.** Baseline Characteristics of Adult Men in 3 Provincial Prisons in Quebec, Canada, 2021

Characteristic	Établissement de détention de Montréal	Établissement de détention de Rivière-des-Prairies	Établissement de détention de Saint-Jérôme	Total
	n = 600 (55%)	n = 300 (27%)	n = 200 (18%)	n = 1100
**Sociodemographic**				
Age, mean (standard deviation), years	39.0 (12.3)	38.2 (12.6)	38.5 (12.8)	38.7 (12.5)
Age category, n (%), years				
18–29	149 (25)	92 (31)	52 (27)	293 (26)
30–39	192 (32)	76 (25)	61 (30)	329 (30)
40–49	136 (23)	66 (22)	49 (24)	251 (23)
≥50	123 (20)	66 (22)	38 (19)	227 (21)
Race/ethnicity, n (%)				
White, non-Hispanic	392 (65)	178 (59)	136 (68)	706 (64)
Black, non-Hispanic	77 (13)	45 (15)	4 (2)	126 (12)
Indigenous	57 (9)	26 (9)	52 (26)	135 (12)
Other visible minority^a^	64 (11)	44 (15)	4 (2)	112 (10)
Missing data	10 (2)	7 (2)	4 (2)	21 (2)
Education level, n (%)				
Less than secondary	211 (35)	107 (35)	85 (43)	403 (37)
Secondary	216 (36)	104 (35)	55 (27)	375 (34)
Post-secondary	172 (29)	87 (29)	50 (25)	309 (28)
Missing data	1 (0.2)	2 (1)	10 (5)	13 (1)
Personal gross yearly income^b^ (USD), n (%)				
$0 or no income	75 (13)	51 (17)	16 (8)	142 (13)
$1–$25,999	228 (38)	122 (41)	76 (38)	426 (39)
$26,000–$55,999	102 (17)	52 (17)	38 (19)	192 (17)
>$56,000	73 (12)	30 (10)	23 (12)	126 (11)
Missing data	122 (20)	45 (15)	47 (23)	214 (20)
Housing status,^c^ n (%)				
Unstable	155 (26)	92 (30)	37 (18)	284 (26)
Stable	415 (69)	203 (68)	151 (76)	769 (70)
Missing data	30 (5)	5 (2)	12 (6)	47 (4)
**Clinical**				
Coronavirus disease 2019 symptoms,^d^ n (%)				
No	328 (55)	160 (53)	116 (58)	604 (55)
Yes	257 (43)	134 (45)	76 (38)	467 (42)
Missing data	15 (2)	6 (2)	8 (4)	29 (3)
Medical comorbidities,^e^ n (%)				
None	299 (50)	152 (51)	103 (52)	554 (50)
One	187 (31)	98 (33)	68 (34)	353 (32)
*>*2	80 (13)	40 (13)	22 (11)	142 (13)
Missing data	34 (6)	10 (3)	7 (3)	51 (5)
Severe acute respiratory syndrome coronavirus 2 serology test result, n (%)				
Negative	436 (73)	256 (85)	162 (81)	854 (78)
Positive	164 (27)	44 (15)	38 (19)	246 (22)
**Carceral**				
Time spent incarcerated since March 2020, n (%)				
Little (<10%)^f^	168 (28)	86 (29)	35 (17)	289 (26)
Some (10%–49%)	162 (27)	81 (27)	88 (44)	331 (30)
Most (50%–99%)	103 (17)	61 (20)	23 (12)	187 (17)
All (100%)	110 (18)	46 (15)	20 (10)	176 (16)
Missing data	57 (10)	26 (9)	34 (17)	117 (11)
Room type, n (%)				
Single cell	128 (21)	30 (10)	15 (7)	173 (16)
Shared cell	460 (77)	266 (89)	181 (91)	907 (82)
Missing data	12 (2)	4 (1)	4 (2)	20 (2)
Employment during incarceration, n (%)				
No	484 (81)	242 (81)	156 (78)	882 (80)
Yes	72 (12)	38 (12)	32 (16)	142 (13)
Missing data	44 (7)	20 (7)	12 (6)	76 (7)
Meal consumption, n (%)				
Alone	190 (32)	81 (27)	36 (18)	307 (28)
Cellmates	143 (24)	78 (26)	19 (10)	240 (22)
Sector	214 (35)	132 (44)	135 (67)	481 (44)
Missing data	53 (9)	9 (3)	10 (5)	72 (6)
Timing of incarceration at screening, n (%)				
Pre-outbreak	130 (22)	0 (0)	106 (53)	236 (22)
Post-outbreak	470 (78)	300 (100)	94 (47)	864 (78)

Other visible minorities include Hispanic, Asian, and Arab.

Personal gross yearly income refers to total annual income (CAD) from all paid work and all other sources before taxes and other deductions in the year prior to incarceration.

Stable housing refers to living in an apartment, condo, or house; unstable housing refers to living in a shelter, group home, hotel, or having no fixed address.

Coronavirus disease 2019 symptoms include fever, chills, headache, sore throat, new or worsening cough, stuffy nose/congestion, difficulty breathing/shortness of breath, loss of smell or taste, fatigue, weakness, confusion, diarrhea, muscle pain, vomiting, and nausea.

Medical comorbidities include hypertension, diabetes, obesity (based on body mass index), asthma, chronic lung disease, chronic heart disease, chronic kidney disease, liver disease, cancer, chronic blood disorder, chronic neurological disorder, immunocompromised (human immunodeficiency virus), and immunocompromised (other).

Reported spending less than 4 weeks incarcerated since March 2020.

### SARS-COV-2 Seropositivity

A total of 246 (22%) participants tested positive with the anti-SARS-CoV-2 serology test: 164 (27%) at EDM, 44 (15%) at EDRDP, and 38 (19%) at EDSJ. Of these, 192 (78%) reported having at least 1 previous SARS-CoV-2 PCR test, with 122 (64%) testing positive and 70 (36%) testing negative. Among the 122, 83 (68%) reported testing only in detention; 41 of 79 (52%) with available incarceration information reported being permanently incarcerated since March 2020. Among the 854 participants who tested negative, 493 (58%) previously underwent SARS-CoV-2 PCR testing and a minority (2%) reported a prior positive test result. A total of 73 (30%) participants with positive serology test results reported no history of COVID-19 symptoms.

Risk factors for SARS-CoV-2 seropositivity identified in univariate analyses are presented in Table 2. In univariate analysis, Black or other visible minority, unstable housing, COVID-19 symptoms, incarceration at EDM, time spent in prison, employment during incarceration, and incarceration post-outbreak were associated with higher SARS-CoV-2 seroprevalence. In the multivariable models examining the causal effect of various carceral exposures on SARS-CoV-2 seropositivity (Table 3), seropositivity increased with time spent incarcerated (“most time”: aPR, 1.47; 95% CI, 1.01–2.12; “all time”: aPR, 2.17; 95% CI, 1.53–3.07), employment during incarceration (aPR, 1.64; 95% CI, 1.28–2.11), shared meal consumption during incarceration (“with cellmates”: aPR, 1.46; 95% CI, 1.08–1.97; “with sector”: aPR, 1.34; 95% CI, 1.03–1.74), and incarceration post-outbreak (aPR, 2.32; 95% CI, 1.69–3.18). The type of room occupied during incarceration (single cell vs shared cell) was not associated with SARS-CoV-2 seropositivity.

**Table 2.** Unadjusted Associations Between the Risk Factors of Interest and Anti–Severe Acute Respiratory Syndrome Coronavirus 2 Seropositivity Among Adult Men in 3 Provincial Prisons in Quebec, Canada, 2021

Characteristic	Prevalence Ratio	95% Confidence Interval
**Sociodemographic**		
Age category, years		
18–29	Reference	Reference
30–39	0.84	0.63–1.13
40–49	0.84	0.61–1.15
≥50	1.04	0.77–1.40
Race/ethnicity		
White, non-Hispanic	Reference	Reference
Black, non-Hispanic	1.55	1.15–2.08
Indigenous	0.96	0.66–1.39
Other visible minority^a^	1.45	1.05–1.99
Education level		
Less than secondary	Reference	Reference
Secondary	1.10	0.85–1.43
Post-secondary	1.07	0.81–1.41
Housing status^b^		
Stable	Reference	Reference
Unstable	1.51	1.21–1.89
**Clinical**		
Coronavirus disease 2019 symptoms^c^		
No	Reference	Reference
Yes	2.93	2.30–3.74
Medical comorbidities^d^		
None	Reference	Reference
1	0.98	0.76–1.25
≥2	1.15	0.84–1.57
Carceral		
Provincial prison		
Établissement de détention de Rivière-des-Prairies	Reference	Reference
Établissement de détention de Montréal	1.86	1.38–2.52
Établissement de détention de Saint-Jérôme	1.30	0.87–1.92
Time spent incarcerated since March 2020		
Little (<10%)^e^	Reference	Reference
Some (10%–49%)	1.38	0.99–1.92
Most (50%–99%)	1.59	1.11–2.28
All (100%)	2.73	1.99–3.74
Room type		
Single cell	Reference	Reference
Shared cell	0.92	0.69–1.22
Employment during incarceration		
No	Reference	Reference
Yes	1·61	1.24–2.08
Meal consumption		
Alone	Reference	Reference
Cellmates	1.26	0.93–1.73
Sector	1.28	0.97–1.67
Timing of incarceration at screening		
Pre-prison outbreak	Reference	Reference
Post-prison outbreak	1.76	1.26–2.47

Other visible minorities include Hispanic, Asian, and Arab.

Stable housing refers to living in an apartment, condo, or house; unstable housing refers to living in a shelter, group home, hotel, or having no fixed address.

Coronavirus disease 2019 symptoms include fever, chills, headache, sore throat, new or worsening cough, stuffy nose/congestion, difficulty breathing/shortness of breath, loss of smell or taste, fatigue, weakness, confusion, diarrhea, muscle pain, vomiting, and nausea.

Medical comorbidities include hypertension, diabetes, obesity (based on body mass index), asthma, chronic lung disease, chronic heart disease, chronic kidney disease, liver disease, cancer, chronic blood disorder, chronic neurological disorder, immunocompromised (human immunodeficiency virus), and immunocompromised (other).

Reported spending less than 4 weeks incarcerated since March 2020.

**Table 3.** Adjusted Associations Between Carceral Exposures of Interest and Anti–Severe Acute Respiratory Syndrome Coronavirus 2 Seropositivity Among Adult Men in 3 Provincial Prisons in Quebec, Canada (2021)

Model	Carceral Exposure	Adjusted Prevalence Ratio	95% Confidence Interval
1^a^	Time spent incarcerated since March 2020		
	Little (<10%)	Reference	Reference
	Some (10%–49%)	1.32	0.95–1.85
	Most (50%–99%)	1.47	1.01–2.12
	All (100%)	2.17	1.53–3.07
2^b^	Room type		
	Single cell	Reference	Reference
	Shared cell	1.03	0.77–1.36
3^c^	Employment during incarceration		
	No	Reference	Reference
	Yes	1.64	1.28–2.11
4^d^	Meal consumption		
	Alone	Reference	Reference
	Cellmates	1.46	1.08–1.97
	Sector	1.34	1.03–1.74
5^e^	Timing of incarceration at screening		
	Pre-prison outbreak	Reference	Reference
	Post-prison outbreak	2.32	1.69–3.18

Adjusted for age, race/ethnicity, education, housing status, provincial prison, and employment status during incarceration.

Adjusted for medical comorbidities, provincial prison, employment status during incarceration, and prison outbreak.

Adjusted for age, education, medical comorbidities, and provincial prison.

Adjusted for coronavirus disease 2019 symptoms, provincial prison, room type, employment status during incarceration, and prison outbreak.

Adjusted for age, medical comorbidities, provincial prison, room type, meal consumption, and employment status during incarceration.

## DISCUSSION

We offer the first description of SARS-CoV-2 seroprevalence in the Canadian incarcerated population and, to date, the largest seroprevalence study to be conducted in a correctional setting. We observed a seroprevalence that was 2-fold higher than in nonvaccinated individuals in Montreal (13.75%) after the second SARS-CoV-2 wave in Quebec [20]. Although our study differs in sampling and population, our findings likely reflect both a high-risk carceral environment and a population that may be at heightened risk in the community through communal or unstable housing and occupational risk. We also found that several modifiable carceral risk factors, such as time spent in prison, employment, shared meal consumption during incarceration, and incarceration post-prison outbreak, were associated with increased seropositivity. These findings have important implications on public health policies and are of utmost importance until COVID-19 vaccine uptake is high in Canadian provincial prisons.

While we observed a relatively high SARS-CoV-2 seroprevalence among incarcerated individuals, it varied among prisons, ranging from 15% to 27%. This variability likely reflects several factors including the type of incarcerated population in each prison. We found that the prison that primarily houses individuals awaiting sentencing (EDRDP) (ie, on remand) had a SARS-CoV-2 seroprevalence (15%) that paralleled the surrounding community, reflecting the high turnover of on-remand individuals, while the seroprevalence at EDM and EDSJ, which house both shorter- and longer-sentenced individuals, was higher, reflecting the additional risk of incarceration. Differences in seroprevalence were also likely due to the timing of recruitment with respect to prison-based and provincial SARS-CoV-2 outbreaks, as another study has shown [17]. Finally, there are important structural differences (size and spatial organization) among prisons and a variable number of correctional employees at each prison, further contributing to differences in overall SARS-CoV-2 seroprevalence.

We found a time-dependent association between duration of incarceration and seropositivity, suggesting that decarceration is an important strategy in preventing SARS-CoV-2 outbreaks in correctional settings. Decarceration consists of the large-scale release of people who pose minimal risk to public safety, the increased use of home confinement, and the non-carceral management of people arrested for minor offenses [27]. Decarceration, when paired with basic preventative measures, is effective in reducing prison-based SARS-CoV-2 transmission [28, 29] and leads to population-level public health benefits [29]. Such benefits, however, depend on appropriate community reintegration assistance at the time of release. Otherwise, individuals may be liberated only to find temporary housing in shelters or communal spaces, which are environments that are equally prone to SARS-CoV-2 outbreaks [23]. This underscores the use of best practices for implementing decarceration as a mitigation strategy by ensuring that conditions that support safe and successful reentry of those decarcerated are met [30]. Decarceration also entails reduction of unnecessary prison admissions. Prosecution for misdemeanors and other minor crimes such as drug possession could be entirely deferred to minimize the overall prison population. These efforts require collaboration from stakeholders across several disciplines and could have a dramatic effect on preventing SARS-CoV-2 outbreaks in prisons.

Given the very high transmission potential in congregate settings, layering multiple preventative interventions is important. Enhanced occupational safety measures for those who are employed during incarceration are needed [31], as is the consideration of strict individual meal consumption when there is ongoing risk for SARS-CoV-2 transmission. Furthermore, while we did not find an increased risk of seropositivity with shared cells, studies have shown that “dormitory housing” is a risk factor for acute SARS-CoV-2 infection [22, 31, 32] and that converting cells into single occupancy may reduce SARS-CoV-2 transmission in correctional settings [33]. In addition, the relatively high (30%) prevalence of asymptomatic infection undermines prison-based surveillance measures that only test symptomatic individuals and their contacts. A shift toward a broad-based testing approach, including mandatory testing on admission, should be considered going forward [6, 32].

Our study also demonstrated that the capacity to implement infection prevention and control measures in correctional settings is limited, underscoring that vaccination is a necessary component of the preventative armamentarium [8]. In fact, a recent study found that the effectiveness of mRNA vaccines in US prisons was equivalent to randomized trials and observational studies [34], highlighting the importance of accelerated, large-scale COVID-19 vaccine rollout in correctional settings. That said, studies have shown that after achieving high vaccine coverage in prisons, ongoing infection prevention and control measures are necessary to prevent future outbreaks in the viral variant era [35], suggesting that a multimodal approach will likely be required going forward.

There are limitations to our study. First, our study sites were not randomly selected but were chosen based on practical considerations including proximity to laboratory facilities in Montreal. This restricted our study population to adult men incarcerated in 3 of 16 provincial prisons, albeit representing almost half of Quebec’s incarcerated male provincial population. Due to security constraints, we used convenience sampling for recruitment. Among individuals approached for participation, almost half (49%) declined study participation. We were not able to collect demographic information for these individuals, which could have informed whether our study population deviates from the overall prison populations, as well as the possibility for selection bias. Furthermore, all 3 study sites had multiple outbreaks during study recruitment. Our results may thus not be generalizable to other facilities that did not experience a SARS-CoV-2 outbreak or to those that primarily house women. Second, we likely underestimated the SARS-CoV-2 seroprevalence for several reasons. We excluded individuals with suspected or confirmed COVID-19 infection as they were in isolation with restricted access. These individuals, however, became eligible to participate following the completion of their isolation. Further, as recruitment occurred longitudinally and not simultaneously at each of the 3 sites, it is possible that infection-induced antibodies waned over time, increasing the possibility for seroreversion. Third, the cross-sectional study design precludes our ability to determine when and where participants acquired SARS-CoV-2 infection, be it in prison or the community. While our questionnaire inquired where previous testing occurred and about test results, various unmeasured variables related to infection were not collected, thereby impacting our inferences. Fourth, information related to carceral conditions, potential exposures, and risk factors were collected from study questionnaires, which may have introduced response biases such as acquiescence, social desirability, and dissent biases. However, the impact of these biases was likely limited with the use of self-administered questionnaires and the inclusion of noncorrectional nurses in the consent process. Fifth, several steps were taken to mitigate biases. We used DAGs to identify confounders to control for while obtaining total effects, and multiple imputation was used to address missing data. Finally, while several studies have attempted to estimate the risk of infection in correctional settings, the majority estimated prevalence of active infection based on PCR testing [6, 32, 36–38], that is, testing that was often symptom-based or reactive post-outbreak, underscoring the contribution of our study to the dearth of seroprevalence data among this vulnerable population. More importantly, very few studies explored carceral risk factors associated with SARS-CoV-2 seropositivity [22, 31, 32], highlighting the important role of our study to policy makers and public health experts.

In conclusion, we found a high prevalence of exposure to SARS-CoV-2 among men in the provincial prison system in Quebec. There were several modifiable carceral factors that were associated with an increased risk of SARS-CoV-2 seropositivity, highlighting the need for a multimodal approach in preventing future outbreaks. Strategies that seek to mitigate prison-based outbreaks should consist of decarceration, individual meal consumption, single cell occupancy, enhanced occupational safety measures, and infection prevention and control measures including vaccination during incarceration.

## Supplementary Data

Supplementary materials are available at *Clinical Infectious Diseases* online. Consisting of data provided by the authors to benefit the reader, the posted materials are not copyedited and are the sole responsibility of the authors, so questions or comments should be addressed to the corresponding author.

ciac031_suppl_Supplementary_Material

## Notes

***Acknowledgments.*** The authors thank all of the seroprevalence study participants, as well as the Directors of Professional Services at the 3 provincial prisons who granted access to the research team during the coronavirus disease 2019 pandemic. The authors also thank those in the respective departments of finance for compensating participants in a timely manner and the many correctional agents who ensured the safety of the research team. Finally, the authors thank the laboratory technicians at Sacré-Coeur Hospital for ensuring the rapid turnaround of test results.

***Financial support.*** This work was supported by the Public Health Agency of Canada through the Sero-Surveillance and Research (COVID-19 Immunity Task Force Initiative) Program (2021-HQ-000103). N. K. is supported by a career award from the Fonds de Recherche Québec*–*Santé (FRQ-S; junior 1). M. M. G. is supported by a Canada Research Chair (tier 2) in Population Health Modeling.

***Potential conflicts of interest.*** N. K. reports research funding from Gilead Sciences, McGill Interdisciplinary Initiative in Infection and Immunity, Canadian Institutes of Health Research, and Canadian Network on Hepatitis C; reports advisory fees from Gilead Sciences, ViiV Healthcare, Merck, and AbbVie; and reports speaker fees from Gilead Sciences, AbbVie, and Merck, all outside of the submitted work. M. M. G. reports an investigator-sponsored research grant from Gilead Sciences Inc and reports contractual arrangements with the World Health Organization, the Institut national de santé publique du Québec, and the Institut d’excellence en santé et services sociaux du Québec, all outside of the submitted work. M. P. C. reports grants from the McGill Interdisciplinary Initiative in Infection and Immunity and from the Canadian Institutes of Health Research; reports personal fees from GEn1E Lifesciences and nplex biosciences, both outside of the submitted work; is the cofounder of Kanvas Biosciences, Inc and owns equity in the company; and has a pending patent for methods for detecting tissue damage, graft versus host disease, and infections using cell-free DNA profiling pending (Cornell reference 9401-01-US Pending) and a pending patent for methods for assessing the severity and progression of severe acute respiratory syndrome coronavirus 2 infections using cell-free DNA (Cornell reference 9561-01-US Pending). J. C. has received research funding from ViiV Healthcare for an investigator initiated study and from Gilead Sciences for clinical trials and reports remuneration for advisory work (ViiV Healthcare, Gilead Sciences, and Merck Canada) outside of the submitted work. All other authors report no potential conflicts. All authors have submitted the ICMJE Form for Disclosure of Potential Conflicts of Interest. Conflicts that the editors consider relevant to the content of the manuscript have been disclosed.
