# Supplementary material for: Seroprevalence and Risk Factors for Severe Acute Respiratory Syndrome Coronavirus 2 Among Incarcerated Adult Men in Quebec, Canada, 2021
Source: Clin Infect Dis. 2022 Jan 17;75(1):e165–73. doi: 10.1093/cid/ciac031 (PMC8807295; doi:10.1093/cid/ciac031)
Supplement: ciac031_suppl_Supplementary_Material [file ciac031_suppl_supplementary_material.docx]

**Supplementary Material**

**Supplementary Table S1.** Dates of SARS-CoV-2 waves in Quebec, Canada (2020–2021).

**Supplementary Table S2.** Dates of SARS-CoV-2 outbreaks across three provincial prisons in Quebec, Canada (2020–2021).

**Supplementary Table S3.** Adjusted associations between carceral exposures of interest and anti-SARS-CoV-2 seropositivity among adult men in three provincial prisons in Quebec, Canada (2021).

**Supplementary Figure S1.** Direct acyclic graphs (DAGs).

**Supplementary Table S1. Dates of SARS-CoV-2 waves in Quebec, Canada (2020–2021).**

| **Waves** | **Start date^1^** | **End date^1^** | **Number of SARS-CoV-2 cases in Quebec^1^** |
| --- | --- | --- | --- |
| First | February 25, 2020 | July 11, 2020 | 56,535 |
| Second | August 23, 2020 | March 20, 2021 | 240,647 |
| Third | March 21, 2021 | July 17, 2021 | 74,787 |
| Fourth | July 18, 2021 | Ongoing (as of September 30, 2021) | 34,834 |

# **Supplementary Table S2. Dates of SARS-CoV-2 outbreaks across three provincial prisons in Quebec, Canada (2020–2021).**

| **Correctional facility** | **Start date** | **End date** | **Number of SARS-CoV-2 cases among inmates^2^** | **Study recruitment** |
| --- | --- | --- | --- | --- |
| Établissement de détention de Montréal | April 20, 2020 | July 7, 2020 | 95 | January 19 to January 27, 2021 and May 12 to July 20, 2021 |
|  | December 23, 2020 | June 3, 2021 | 171 |  |
| Établissement de détention de Rivière-des-Prairies | December 21, 2020 | April 30, 2021 | 32 | February 3 to March 24, 2021 |
|  | June 19, 2021 | July 7, 2021 | 3 |  |
| Établissement de détention de Saint-Jérôme | January 21, 2021 | February 25, 2021 | 92 | March 30 to April 14, 2021 and September 7 to 15, 2021 |
|  | April 16, 2021 | May 6, 2021 | 2 |  |

# **Supplementary Table S3. Adjusted associations between carceral exposures of interest and anti-SARS-CoV-2 seropositivity among adult men in three provincial prisons in Quebec, Canada (2021).**

| **Models** | **Variables** | **Adjusted Prevalence Ratio (aPR)** | **95% Confidence Interval (CI)** |
| --- | --- | --- | --- |
| Model 1 | **Age category** | *Reference* | |
|  | 18–29 |  |  |
|  | 30–39 | 0.85 | 0.63–1.14 |
|  | 40–49 | 0.84 | 0.61–1.15 |
|  | ≥50 | 1.08 | 0.79–1.48 |
|  | **Race/ethnicity** |  |  |
|  | White, non-Hispanic | *Reference* | |
|  | Black, non-Hispanic | **1.49** | **1.09–2.04** |
|  | Indigenous | 1.02 | 0.72–1.46 |
|  | Other visible minority | **1.44** | **1.05–1.98** |
|  | **Education level** |  |  |
|  | Less than secondary | *Reference* | |
|  | Secondary | 1.08 | 0.84–1.40 |
|  | Post-secondary | 1.11 | 0.84–1.47 |
|  | **Housing status** |  |  |
|  | Stable | *Reference* | |
|  | Unstable | **1.29** | **1.02–1.63** |
|  | **Provincial prison** |  |  |
|  | EDRDP | *Reference* | |
|  | EDM | **1.91** | **1.42–2.58** |
|  | EDSJ | **1.53** | **1.03–2.29** |
|  | **Time spent incarcerated since March 2020** | |  |
|  | Little (<10%) | *Reference* | |
|  | Some (10–49%) | 1.32 | 0.95–1.85 |
|  | Most (50–99%) | **1.47** | **1.01–2.12** |
|  | All (100%) | **2.17** | **1.53–3.07** |
|  | **Employment during incarceration** |  |  |
|  | No | *Reference* | |
|  | Yes | **1.34** | **1.03–1.75** |
| Model 2 | **Medical comorbidities** |  |  |
|  | None | *Reference* | |
|  | 1 | 0.97 | 0.76–1.24 |
|  | ≥2 | 1.05 | 0.78–1.43 |
|  | **Provincial prison** |  |  |
|  | EDRDP | *Reference* | |
|  | EDM | **2.15** | **1.57–2.93** |
|  | EDSJ | **1.82** | **1.24–2.68** |
|  | **Room type** |  |  |
|  | Single cell | *Reference* | |
|  | Shared cell | 1.03 | 0.77–1.36 |
|  | **Employment during incarceration** | | |
|  | No | *Reference* | |
|  | Yes | **1.78** | **1.39–2.28** |
|  | **Timing of incarceration at screening** | | |
|  | Pre-outbreak | *Reference* | |
|  | Post-outbreak | **2.20** | **1.60–3.02** |
| Model 3 | **Age category** |  |  |
|  | 18–29 | *Reference* | |
|  | 30–39 | 0.79 | 0.59–1.07 |
|  | 40–49 | 0.79 | 0.57–1.09 |
|  | ≥50 | 0.97 | 0.71–1.34 |
|  | **Education level** |  |  |
|  | Less than secondary | *Reference* | |
|  | Secondary | 1.09 | 0.84–1.41 |
|  | Post-secondary | 1.09 | 0.82–1.45 |
|  | **Medical comorbidities** |  |  |
|  | None | *Reference* | |
|  | 1 | 0.96 | 0.75–1.23 |
|  | ≥2 | 1.09 | 0.79–1.49 |
|  | **Provincial prison** |  |  |
|  | EDRDP | *Reference* | |
|  | EDM | **1.90** | **1.40–2.56** |
|  | EDSJ | 1.30 | 0.88–1.92 |
| **Employment during incarceration** | | | |
|  | No | *Reference* | |
|  | Yes | **1.64** | **1.28–2.11** |
| Model 4 | **COVID-19 symptoms** |  |  |
|  | No | *Reference* | |
|  | Yes | **2.80** | **2.20–3.56** |
|  | **Provincial prison** |  |  |
|  | EDRDP | *Reference* | |
|  | EDM | **2.15** | **1.59–2.91** |
|  | EDSJ | **1.91** | **1.30–2.82** |
|  | **Room type** |  |  |
|  | Single cell | *Reference* | |
|  | Shared cell | 0.85 | 0.64–1.12 |
|  | **Employment during incarceration** | | |
|  | No | *Reference* | |
|  | Yes | **1.64** | **1.28–2.11** |
|  | **Meal consumption** |  |  |
|  | Alone | *Reference* | |
|  | Cellmates | **1.46** | **1.08–1.97** |
|  | Sector | **1.34** | **1.03–1.74** |
|  | **Timing of incarceration at screening** | |  |
|  | Pre-outbreak | *Reference* | |
|  | Post-outbreak | **2.21** | **1.62–3.00** |
| Model 5 | **Age category** |  |  |
|  | 18–29 | *Reference* | |
|  | 30–39 | 0.79 | 0.60–1.06 |
|  | 40–49 | 0.85 | 0.62–1.16 |
|  | ≥50 | 1.00 | 0.73–1.36 |
|  | **Medical comorbidities** |  |  |
|  | None | *Reference* | |
|  | 1 | 0.95 | 0.74–1.20 |
|  | ≥2 | 1.00 | 0.73–1.38 |
|  | **Provincial prison** |  |  |
|  | EDRDP | *Reference* | |
|  | EDM | **2.21** | **1.62–3.01** |
|  | EDSJ | **1.86** | **1.26–2.76** |
|  | **Room type** |  |  |
|  | Single cell | *Reference* | |
|  | Shared cell | 0.88 | 0.65–1.18 |
|  | **Employment during incarceration** | | |
|  | No | *Reference* | |
|  | Yes | **1.79** | **1.38–2.33** |
|  | **Meal consumption** |  |  |
|  | Alone | *Reference* | |
|  | Cellmates | **1.50** | **1.09–2.07** |
|  | Sector | **1.45** | **1.11–1.91** |
|  | **Timing of incarceration at screening** | |  |
|  | Pre-outbreak | *Reference* | |
|  | Post-outbreak | **2.32** | **1.69–3.18** |

aPR: adjusted prevalence ratio; CI: confidence interval.

**
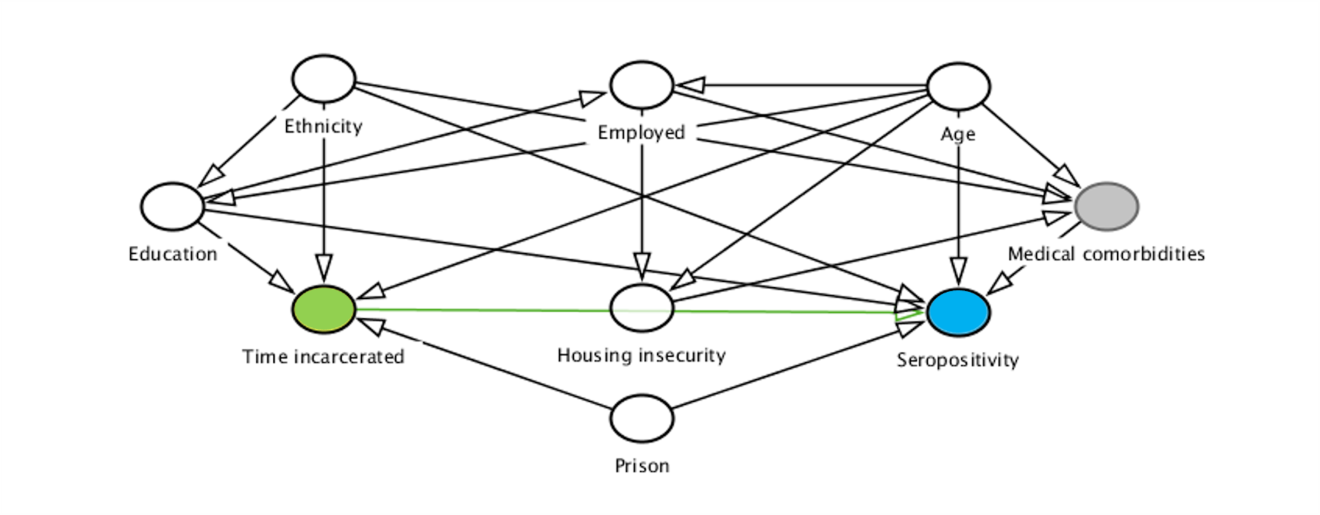
**

a.

**
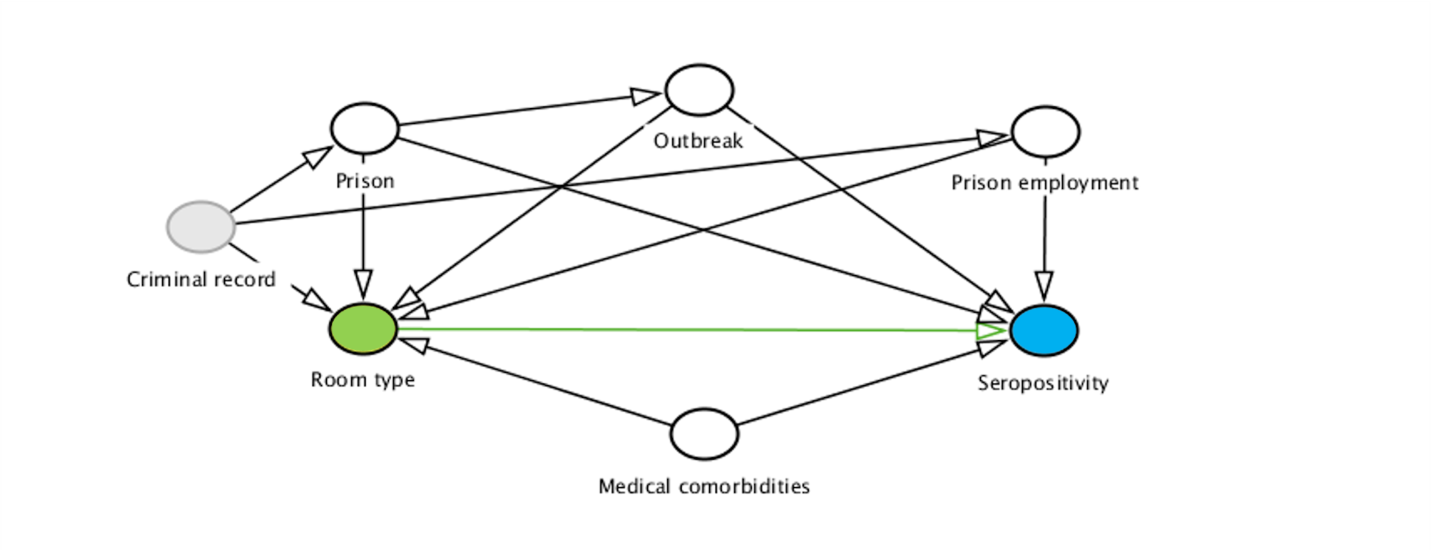
**

c.

b.

d.

**
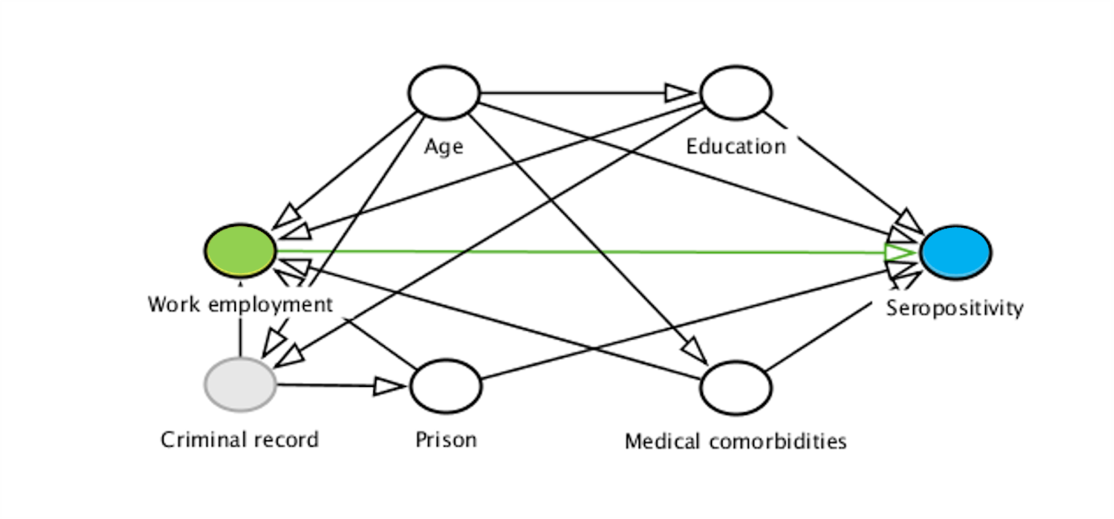
**

**
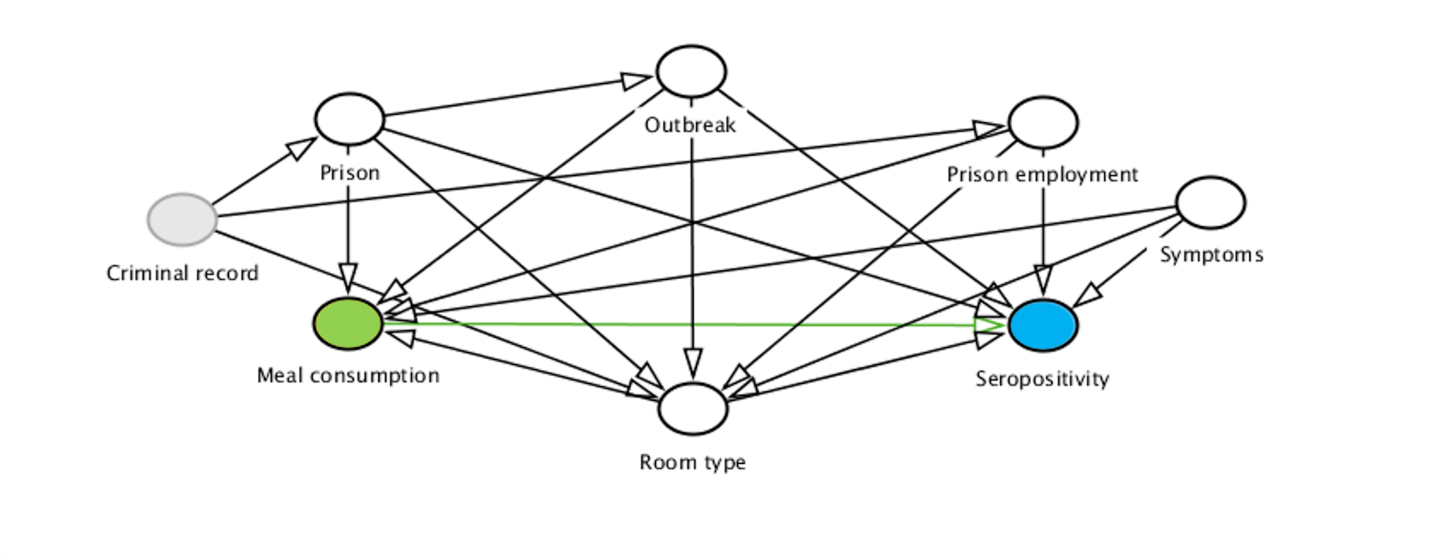
**

**
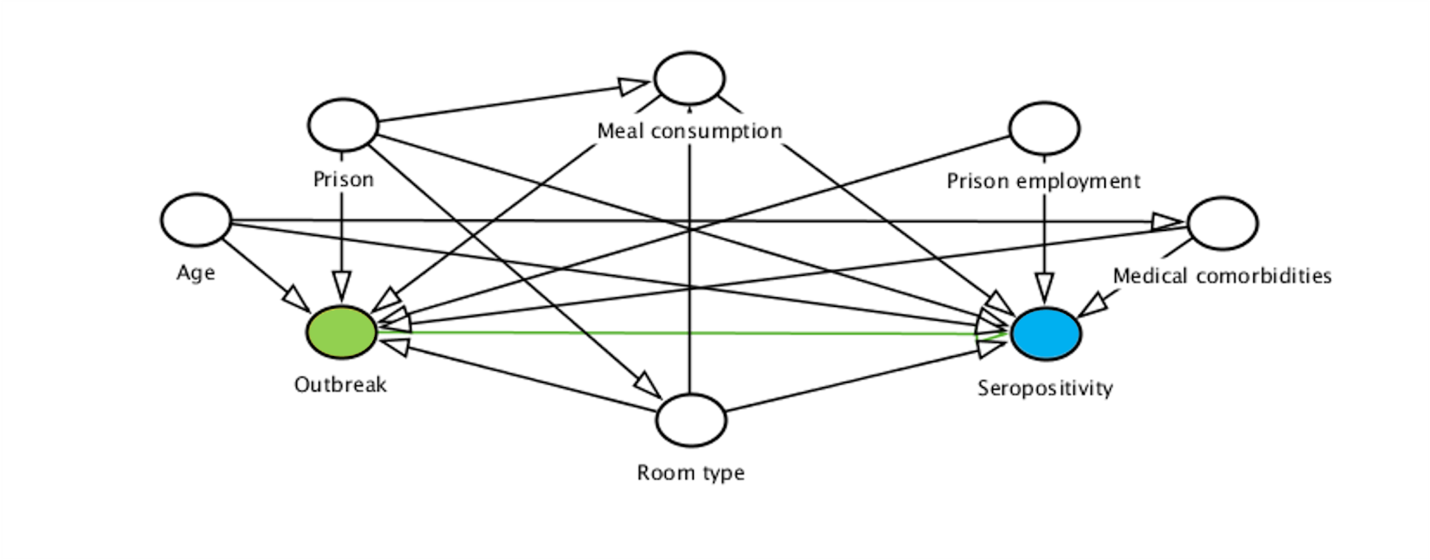
**

e.

**
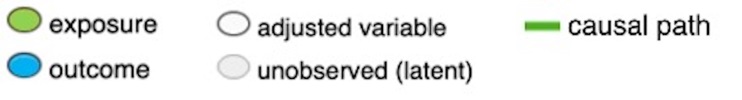
**

**Supplementary Figure S1. Direct acyclic graphs (DAGs).**

Directed acyclic graphs depicting known or plausible relationship between the carecral exposure of interest on SARS-CoV-2 seropositivity. a. Time spent incarcerated since March 2020, adjusting for age, race/ethnicity, education, housing status, provincial prison, and employment status during incarceration (Model 1); b. Room type, adjusting for medical comorbidities, provincial prison, employment status during incarceration, and timing of incarceration at screening (Model 2); c. Employment during incarceration, adjusting for age, education, medical comorbidities, and provincial prison (Model 3); d. Meal consumption during incarceration, adjusting for COVID-19 symptoms, provincial prison, room type, employment status during incarceration, and timing of incarceration at screening (Model 4); e. Timing of incarceration at screening, adjusting for age, medical comorbidities, provincial prison, room type, meal consumption, and employment status during incarceration (Model 5).

**References**

1. Institut national de santé publique du Québec. Ligne du temps COVID-19 au Québec. October 7, 2021. Accessed October 15, 2021. <https://www.inspq.qc.ca/covid-19/donnees/ligne-du-temps>
2. Gouvernement du Québec. Nombre de cas dans les établissements de détention. September 29, 2021. Accessed September 30, 2021. <https://www.quebec.ca/sante/problemes-de-sante/a-z/coronavirus-2019/situation-coronavirus-quebec/#c57309>
